# Data for transcriptomic and proteomic analyses of leaves from *Clematis terniflora* DC. under binary stress

**DOI:** 10.1016/j.dib.2017.03.021

**Published:** 2017-03-25

**Authors:** Bingxian Yang, Qijie Guan, Jingkui Tian, Setsuko Komatsu

**Affiliations:** a College of Biomedical Engineering & Instrument Science, Zhejiang University, Hangzhou 310027, China; b National Institute of Crop Science, National Agriculture and Food Research Organization, Tsukuba 305-8518, Japan

**Keywords:** Transcriptomics, Proteomics, *Clematis terniflora* DC

## Abstract

High level of UV-B irradiation followed by dark treatment accumulates secondary metabolites in *Clematis terniflora* DC. To investigate the response mechanism under high level of UV-B irradiation followed by dark treatment, transcriptomic and proteomic analyses were performed in leaves of *Clematis terniflora* DC. The experimental design for the transcriptomic and proteomic analyses in leaves of *C. terniflora* under stresses was organized into a picture. For transcriptomics, mRNA-sequencing technology was used. Genes identified in leaves of *C. terniflora* at starting point, high level of UV-B irradiation, and high level of UV-B irradiation followed by dark treatment were listed; genes with different expression levels at starting point, high level of UV-B irradiation, and high level of UV-B irradiation followed by dark treatment were also presented in this DiB article. For proteomics, a gel-free/label-free proteomic technique was used. Proteins with different abundances in leaves at starting point, high level of UV-B irradiation, and high level of UV-B irradiation followed by dark treatment were presented in this DiB article. In order to monitor the expression levels of genes under the stress, quantitative reverse transcription polymerase chain reaction was performed. The primer sequences of genes selected for quantitative reverse transcription polymerase chain reaction was presented in this DiB article.

**Specifications Table**

**Table:** 

Subject area	Biology, Psychology
More specific subject area	Transcriptomics, Proteomics
Type of data	Table, text file, figure
How data was acquired	*mRNA-sequencing, mass spectroscopy*
Data format	*Filtered, Analyzed*
Experimental factors	*High level of UV-B irradiation, dark treatment*
Experimental features	*High level of UV-B irradiation for 5 h followed by dark treatment for 48 h*
Data source location	*Tsukuba Japan, Hangzhou China*
Data accessibility	*Data are available with this article*

**Value of the data**

• These data could supply a comprehensive understanding in revealing the induced response of *C. ternilora* at transcriptome and proteome levels [1].
• Transcriptomic and proteomic technologies used in this article revealed the response mechanisms in leaves of *C. terniflora* to high level of UV-B irradiation followed by dark treatment.
• Because of loose correlation between mRNA and protein, the integration of transcriptomics and proteomics are useful for functional analysis of plant under UV-B irradiation followed by dark treatment.

## 1 Data

**Fig. 1:**
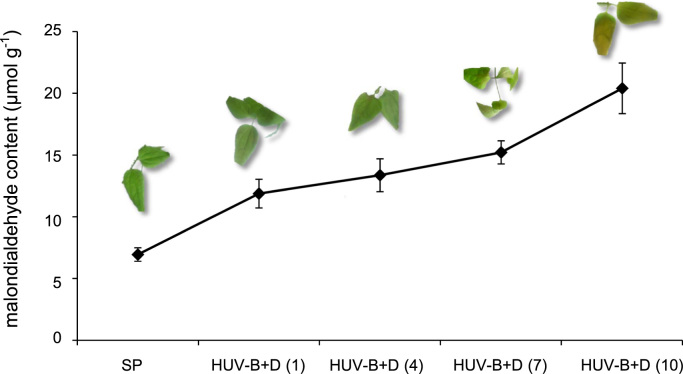
Morphological characteristics of leaves of *C. terniflora* under high level of UV-B irradiation followed by dark treatment. Number in parenthesis indicates the time of doses of UV-B irradiation than 15 kJ m^−2^ d^−1^. Malondialdehyde content was determined and the data are shown as the mean±SD from three independent biological replicates. The photographs show the condition of leaves of *C. terniflora* for each treatment condition.

**Fig. 2:**
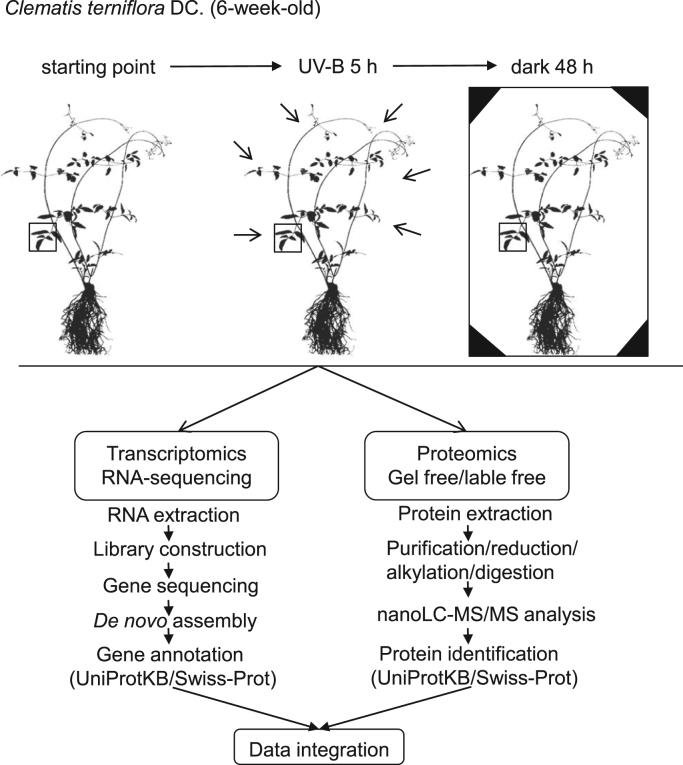
Experimental design for the transcriptomic and proteomic analyses in leaves of *C. terniflora* under high level of UV-B irradiation followed by dark treatment. Six-week-old *C. terniflora* plant was irradiated with high level UV-B for 5 h and treated with dark for 48 h. Leaves were collected from plants at each treatment, mRNAs/proteins were extracted, and transcriptomic and proteomic analyses were performed. Three independent biological replicates were employed. Data were integrated to manifest a comprehensive understanding on the perturbation of the metabolic pathways.

The malondialdehyde content in leaves of *C. terniflora* under different doses of UV-B irradiation was measured (Fig. 1). The experimental design for the proteomic and transcriptomic analyses in leaves of *C. terniflora* under stresses was organized into a picture and presented in the DiB article (Fig. 2). The primer sequences of genes selected for quantitative reverse transcription polymerase chain reaction were presented in this DiB article (Table 1). Genes identified in leaves of *C. terniflora* at starting point (Table 2), high level of UV-B irradiation (Table 3), and high level of UV-B irradiation followed by dark treatment (Table 4) were presented. Genes with different expression levels at starting point, high level of UV-B irradiation, and high level of UV-B irradiation followed by dark treatment (Tables 5 and 6) were presented in this DiB article. Proteins with different abundances in leaves at starting point, high level of UV-B irradiation, high level of UV-B irradiation followed by dark treatment (Tables 7 and 8) were also presented in this DiB article. Furthermore, the original proteomic data exported from proteome discoverer have been attached (starting point, Supplemental Tables 1–3; UV-B, Supplemental Tables 4–6; UV-B+D, Supplemental Tables 7–9).

## 2 Experimental design, materials and methods

### 2.1 Plant material and treatments

Seeds of *Clematis terniflora* DC. were incubated in water and the germinated seeds were sown into seedbeds. The seedlings were transplanted into potted containers and placed in greenhouse, which was controlled at 28–30 °C, 70–80% relative humidity, and 160 µmol m^−2^ s^−1^ of white light irradiance. After 6 weeks, plants were treated with UV-B irradiation in a cabinet, which was controlled at 25–30 °C, 80% relative humidity. The intensity of UV-B irradiation on the surface was at 104.4 kJ m^−2^ d^−1^, which was measured by a UV Light Meter (Beijing Normal University, Beijing, China). After UV-B irradiation, plants were treated with dark for 48 h. Leaves in the area from the basal 10–60 cm in each plant were collected for transcriptomic, proteomic, phytochemical, and enzymatic analyses. Five leaves were collected for one replication and 3 independent biological replicates were performed. Independent biological replicates mean that plants were treated with different date.

### 2.2 Proteomic analysis

Proteins were extracted, reduced, alkylated, digested, and analyzed by nanoLC-MS/MS. Peptide ions were analyzed on a nanospray LTQ Orbitrap MS (Thermo Fisher Scientific, San Jose, CA, USA) operated in data-dependent acquisition mode with the installed Xcalibur software (version 2.1; Thermo Fisher Scientific). The acquired MS spectra were used for protein identification. Proteins were identified using the Mascot search engine (version 2.5.1; Matrix Science, London, UK) with UniProtKB/Swiss-Prot database (Viridiplantae; Release 2015_11, 37,036 sequences, http://www.uniprot.org/uniprot/). DTA files were generated from acquired raw data files and then converted to Mascot generic files using Proteome Discoverer software (version 1.4.0.288; Thermo Fisher Scientific). Trypsin was specified as the proteolytic enzyme and one missed cleavage was allowed. Peptide mass tolerance was set at 10 ppm, fragment mass tolerance was set at 0.8 Da, and peptide charges were set at +2, +3, and +4. An automatic decoy database search was performed as part of the search. Mascot data were filtered with the Mascot percolator to improve the accuracy and sensitivity of peptide identification [2]. False discovery rates for peptide identification of all searches were less than 1.0%. Peptides with a percolator ion score of more than 13 and a *p*-value less than 0.05 were used for protein identification.

The Mascot search data were exported in msf format for SIEVE analysis (version 2.1.377; Thermo Fisher Scientific). The relative abundances of peptides and proteins were compared between samples. For the analysis, the chromatographic peaks detected by MS were aligned and the peptide peaks were detected as a frame. Chromatographic peak areas of each sample within a single frame were compared and the ratios between samples in each frame were determined. The frames detected in the MS/MS scan were matched to the imported Mascot search data. The ratio of peptides between samples was determined from the relative variance-weighted average of the ratios in frames, which matched the peptides in the MS/MS spectrum. The ratios of peptides were further integrated to determine the ratio of the corresponding protein. In the differential analysis of protein abundance, total ion current was used for normalization. The minimum requirement for the identification of a protein was a minimum of 2 matched peptides and a *p*-value less than 0.05.

### 2.3 Transcriptomic analysis

RNAs were extracted, sequenced, and analyzed. The mRNA-sequencing libraries were sequenced using a paired end read protocol with 100 base pairs of data collected per run on the Hiseq. 2000 sequencing platform (Illumina). *De novo* assembly of the transcripts was carried out using the short read assembly program ‘Trinity’ [3], [4]. Unigene sequences were aligned by BLASTX to UniProtKB/Swiss-Prot database using an E-value cut-off of 10^−5^. Coding sequence regions were determined for the highest-ranked proteins using BLAST. Unigenes that could not be aligned to database were scanned by ESTScan [5] to determine the nucleotide (5′−3′) and amino acid sequences of the coding regions. The value of reads per kilobase million (RPKM) was used to represent the expression level of genes. For gene identification analysis, the alternatively spliced genes were filed according to the criterion which is more than 100 by the RPKM value of transcripts [6]. For gene expression analysis, all the genes with RPKM value were used. A significant change of gene expression between samples is identified as 10-fold change of RPKM value.

### 2.4 Functional annotation

Protein and gene functions were categorized using MapMan bin codes (http://mapman.gabipd.org/) [7]. The predication of identified proteins and genes derived from *C. terniflora* was performed by transferring annotations to the Arabidopsis genome and consideration of orthologous genes.
